# Barriers to early presentation of breast cancer among women in Soweto, South Africa

**DOI:** 10.1371/journal.pone.0192071

**Published:** 2018-02-02

**Authors:** Maureen Joffe, Oluwatosin Ayeni, Shane Anthony Norris, Valerie Ann McCormack, Paul Ruff, Ishani Das, Alfred I. Neugut, Judith S. Jacobson, Herbert Cubasch

**Affiliations:** 1 Non-Communicable Diseases Research Division, Wits Health Consortium (PTY) Ltd, Johannesburg, South Africa; 2 MRC Developmental Pathways to Health Research Unit, Department of Pediatrics, Faculty of Health Sciences, University of Witwatersrand, Johannesburg, South Africa; 3 Section of Environment and Radiation, International Agency for Research on Cancer, Lyon, France; 4 Division of Medical Oncology, Department Internal Medicine, University of Witwatersrand Faculty of Health Sciences, Johannesburg, South Africa; 5 Herbert Irving Comprehensive Cancer Center, Columbia University, New York, NY, United States of America; 6 Department of Medicine, College of Physicians and Surgeons, Columbia University, New York, NY, United States of America; 7 Department of Epidemiology, Mailman School of Public Health, Columbia University, New York, NY, United States of America; 8 Department of Surgery, Faculty of Health Sciences, University of Witwatersrand, Johannesburg, South Africa; Iranian Institute for Health Sciences Research, ISLAMIC REPUBLIC OF IRAN

## Abstract

**Purpose:**

Reported breast cancer incidence is rising in South Africa, where some women are diagnosed late and have poor outcomes. We studied patient and provider factors associated with clinical stage at diagnosis among women diagnosed at the Chris Hani Baragwanath Academic Hospital in Soweto, Johannesburg in 2015–2016.

**Methods:**

From face-to-face interviewer-administered questionnaires we compared self-reported socioeconomics, demographics, comorbidities, risk factors, personal and health system barriers, and from patient clinical records, clinical staging, receptor subtype, and tumor grade among 499 consecutive women newly diagnosed with advanced stage (III/IV) breast cancer versus those diagnosed early (stage 0/I/II). Logistic regression models were used to identify factors associated with advanced stage at diagnosis.

**Results:**

Among the women, 243 (49%) were diagnosed at early and 256 (51%) at advanced stages. In the multiple logistic regression adjusted model, completion of high school or beyond (odds ratio (OR) 0.59, and greater breast cancer knowledge and awareness (OR 0.86) were associated with lower stage of breast cancer at presentation. Advanced stage was associated with Luminal B (OR 2.25) and triple-negative subtypes (OR 3.17) compared to luminal A, with delays >3 months from first breast symptoms to accessing the health system (OR 2.79) and with having more than 1 visit within the referral health system (OR 3.19) for 2 visits; OR 2.73 for ≥3 visits).

**Conclusions:**

Limited patient education, breast cancer knowledge and awareness, and health system inefficiencies were associated with advanced stage at diagnosis. Sustained community and healthcare worker education may down-stage disease and improve cancer outcomes.

## Introduction

Breast cancer is the most common cancer among women and a major cause of cancer deaths in women worldwide [1, 2]. Although breast cancer was once considered a disease of affluence, its reported incidence is rising rapidly in low- and middle-income countries (LMICs), such as South Africa (SA). In recent years, with the expanded ARV rollout, SA’s HIV control and life expectancy have dramatically improved, whilst its non-communicable disease burden is growing[1, 3, 4]. An upper middle-income country (UMIC), with an annual per capita Purchasing Power Parity (PPP) gross domestic product of approximately US$13,500[5, 6], SA has a better developed healthcare system than most sub-Saharan African countries. Its private sector facilities are comparable to those of high income countries (HICs), and its public sector facilities, serving approximately 85% of the population, include a number of relatively well equipped and staffed academic hospitals.

The published age-standardized annual breast cancer incidence rate (ASR) per 100,000 is 29.1 among black SA women, much lower than the rates among mixed race, Asian and white SA women (52.92–79.3), and amongst USA black (125.1) and white women (127.7) [1, 7–10]. Yet SA breast cancer mortality rates among socioeconomically disadvantaged patients, although poorly reported, are assumed to be high [11, 12]. This is partly due to more than 70% of patients being diagnosed with advanced stage disease in rural and urban health care settings [3, 13–16]. In the USA, black women also have higher breast cancer-specific mortality rates than do white women [17]. Younger age at presentation and greater proportions of more aggressive breast cancer subtypes are also thought to contribute to those higher mortality rates. [8, 9, 14, 17–21]. Yet Johannesburg and South African National Cancer Registry (SANCR) data reveal that, like USA women, more than 60% of SA women, regardless of race, are diagnosed with estrogen receptor-positive (ER+) tumors [14, 21]. Hence outcomes should improve among SA women if they do not encounter delays in their diagnosis and treatment.

Despite the large proportion of women who present with late stage disease in SA, we observed an encouraging down-staging of invasive breast cancer diagnosed at the Chris Hani Baragwanath Academic Hospital (CHBAH) in Soweto, Johannesburg, between 2007 and 2012. The proportion of stage I and II cancers increased over that time interval from approximately 30% to more than 50% [22]. We attributed this improvement to the implementation of an open access clinic and successful breast cancer awareness campaigns. But we still fall well short of the 80–90% proportion of early-stage disease reported for HICs[2]. The personal, societal and health system barriers that impact stage at diagnosis are not well understood in our setting. It is hoped that modifiable factors identified may be addressed in future intervention studies applicable to urban patients treated at CHBAH and to disadvantaged patients from other regions of the country. Prior studies from LMICs and HICs suggest that various patient and health system factors may affect the timeliness of breast cancer diagnosis [4, 23–32]. To inform future interventional work, we undertook a study of socio-demographic, clinical, lifestyle, and health system factors associated with advanced-stage diagnosis of breast cancer at CHBAH.

## Methods

### Study population

CHBAH is a public academic hospital located in Soweto, in the southwestern part of Johannesburg, South Africa. It serves as the referral facility for approximately 3 million people who live within a 60 km radius of the hospital, as well as for patients from farther afield.

In SA’s hierarchical referral system, doctors and nurses at primary care clinics refer patients with breast conditions either directly to an academic tertiary hospital or to a local secondary hospital, which can provide general hospital care and some diagnostic services and can refer patients who need more advanced care to a tertiary hospital. CHBAH has a specialized Surgical Breast Unit where currently 25–35 patients per month are diagnosed with breast cancer.

### Recruitment and data collection

In this cross-sectional survey, from January 8, 2015, through December 31, 2016, at the CHBAH, 499 consecutive female patients who were 18+ years and newly diagnosed with stage 0-IV breast carcinoma completed an informed, written consent and were enrolled into our study. We had no refusals to participate; therefore our study represents all women who presented at the clinic during this time period. Self-reported socioeconomic and demographic variables were collected, namely date of birth from which age at diagnosis was determined, marital status, highest education level completed, household possession score (determined from home ownership, car ownership, microwave, washing machine, indoor running water, flush toilet inside home with calculations described in the legend to Table 1), employment status and parity (full-term pregnancies). Lifestyle risk factors (BMI, calculated from weight and height measurements, self-reported alcohol consumption, smoking, hypertension, diabetes, and cardiovascular disease) and clinical data (HIV status measured by the ELISA test, intrinsic breast cancer subtypes, grade, and clinical staging) were recorded. Intrinsic breast cancer subtyping included Allred scoring for definition of estrogen and progesterone receptor (ER and PR) status [33]. Luminal A subtypes were defined as ER / PR+ HER2-negative and Ki67 ≤15%; Luminal B as ER/ PR+ HER2-positive and/or Ki67 >15%; HER2-enriched as ER/PR- and HER2- positive; and triple negative as ER/PR- and HER2-negative.

**Table 1:** Comparison of the socio-demographic, clinical and lifestyle characteristics, knowledge and awareness of breast cancer and health system barriers among patients diagnosed with early and late stage disease at the CHBAH breast clinic.

	Total	Early stage	Late stage	ρ-	Chi square
		(0-II)	(III & IV)	value	Statistic/DF
	N = 499(%)	N = 243 (%)	N = 256 (%)		
**SOCIO-DEMOGRAPHIC CHARACTERISTICS**					
**Age**					
<40	69 (13.8)	23 (9.4)	46 (18.0)	**0.013**	12.6788
40–49	124 (24.8)	58 (23.9)	66 (25.8)		DF = 4
50–59	120 (24.1)	59 (24.3)	61 (23.8)		
60–69	102 (20.4)	62 (25.5)	40 (15.6)		
70 and above	84 (16.8)	41(16.9)	43 (16.8)		
**Marital status**					
Single	119 (24.0)	55 (22.7)	64 (25.2)	0.736	0.6133
Married/co-habiting	216 (43.6)	105 (43.4)	111 (43.7)		DF = 2
Divorced/widowed	161 (32.4)	82 (33.9)	79 (31.1)		
**Level of education**					
Completion of Informal / Primary	142 (28.5)	56 (23.1)	86 (33.6)	**0.010**	6.6688
Completion of High school/ any tertiary education	348 (71.5)	186 (76.9)	170 (66.4)		DF = 1
**Household socioeconomic status** ^**a**^					
Mean ± S.D*	3.71 ± 1.63	3.88 ± 1.65	3.54 ± 1.60	**0.0226**	Test stat
					2.287
					DF = 497
**Employment status**					
Unemployed	229 (45.9)	118 (48.5)	111 (43.4)	0.244	2.8184
Employed	136 (27.2)	58 (23.9)	78 (30.5)		DF = 2
Retired	134 (26.9)	67 (27.6)	67 (26.1)		
**Parity**^**b**^ (Mean ± S.D.)	3.00 ± 1.84	2.84 ± 1.68	3.15 ± 1.97	**0.064**	t = -1.8596
					DF = 469
**CLINICAL & LIFESTYLE FACTORS**					
**BMI**^**b**^ (Mean ±S.D.)	31.42 ± 7.85	31.77 ± 6.98	31.08 ± 8.63	**0.337**	0.9645
					DF = 468
**Alcohol consumption**					
Yes	100 (20.0)	50 (20.6)	50 (19.5)	0.771	0.0849
No	399 (80.0)	193 (79.4)	206 (80.5)		DF = 1
**Smoking**					
Yes	35 (7.0)	21 (8.6)	14 (5.5)	0.165	1.9247
No	464 (93.0)	222 (91.4)	242 (94.5)		DF = 1
**Hypertension**					
Yes	200 (40.3)	113 (46.7)	87 (34.3)	**0.005**	7.9727
No	296 (59.7)	129 (53.3)	167 (65.7)		DF = 1
**HIV Status**					
Positive	113 (22.7)	44 (18.9)	69 (28.6)	**0.013**	6.1983
Negative	361 (72.3)	189 (81.1)	172 (71.4)		DF = 1
Unknown	25	10	15		
**Receptor subtype–intrinsic**					
Luminal A	54 (10.9)	31 (12.9)	23 (9.0)	0.276	3.8707
Luminal B	350 (70.6)	171 (71.0)	179 (70.2)		DF = 3
HER2 enriched	33 (6.6)	16 (6.6)	17 (6.7)		
TRIPLE NEGATIVE	59 (11.9)	23 (9.5)	36 (14.1)		
**Grade**					
Low/intermediate	275 (59.0)	133 (60.2)	142 (58.0)	0.626	0.2371
High	191 (41.1)	88 (39.8)	103 (42.0)		DF = 1
Unknown	33	22	11		
**KNOWLEDGE (score 0–9)**^c^					
Mean ± S.D	5.86 ± 1.88	6.20 ± 1.73	5.53 ± 1.97	**0.0001**	DF = 497
**HEALTH SYSTEM FACTORS** **Prior clinical breast examination**					
None/Self	481 (96.4)	233 (95.9)	248 (96.9)	0.553	0.3516
C**l**inician	18 (3.6)	10 (4.1)	8 (3.1)		DF = 1
**Clinical waiting times as a barrier**					
Yes	42 (8.4)	23 (9.5)	19 (7.4)	0.411	0.675
No	457 ((1.6)	220 (90.5)	237 (92.6)		DF = 1

^a^HOUSEHOLD SOCIO-ECONOMIC STATUS (possessions) score* determined from: (Home ownership = 1, Car ownership = 1, Microwave = 1, Washing machine = 1, Indoor running water = 1, Flush toilet inside home = 1. Denominator = 6 (1 for Yes, 0 for No)

^b^Analyzed as continuous variables DF = Degrees of freedom.

^c^SELF-REPORTED KNOWLEDGE/ AWARENESS OF BREAST CANCER: 1 was allocated for every correct answer, 0 for wrong answer or don’t know for each of 9 questions probing knowledge of breast cancer and symptoms.

The University of the Witwatersrand Human Research Ethics Committee (HREC) Medical approved the study (M141102, dated January 7, 2015. Clinical data were extracted from the electronic clinical records in the CHBAH Breast Unit and linked to the Barriers to Care questionnaire described below. The anonymized data set used for the analysis is provided as supporting information, S1 Table).

### Barriers to care questionnaire

The Barriers to Care questionnaire was developed from a literature review on barriers to early-stage presentation of breast cancer among women from LMICs (which referenced validated survey instruments for HICs), considering specific parameters unique to CHBAH. Key themes emerged around awareness of breast cancer and its risk factors[34–39], attitudes toward breast cancer and health-seeking behavior[40–46] and personal and health system barriers to accessing care[47–60]. Two focus group sessions, each comprising 15 former breast cancer patients were undertaken. They reiterated the emergent themes from the literature and supported their relevance to our Soweto urban setting. Ten research and nursing staff then developed the 24 item questionnaire that covered 8 major domains, namely awareness and knowledge of breast cancer (9 questions); personal hurdles/barriers to seeking help (2 questions); family hurdles (3 questions); community hurdles (1 question); cultural hurdles (2 questions); economic hurdles (4 questions); and health system barriers to care (3 questions).Yes, no and unsure were response options to questions asked and a five point agreement Likert Scale (strongly disagree, disagree, unsure/neutral, agree, strongly agree) was used to measure respondents' agreement with statements used in the questionnaire (provided as supplementary information, S1 Text). The knowledge and awareness score (0–9) was calculated from summations of 1 and 0 values assigned respectively for yes and no answers to questions and for correct and incorrect agreements to statements (irrespective of strength of agreement). The participant pathways in the referral health system were probed in detail covering choice of initial consultation, referral within the primary and secondary healthcare facilities, and the number of visits experienced prior to reaching the CHBAH Breast Clinic. We queried time intervals between symptom awareness and visits to healthcare facilities for diagnosis and treatment and between all visits prior to reaching CHBAH, and asked about personal reasons for delays longer than 1 month. We also probed causes for delays within the referral network. We evaluated the understanding of each question by the research team and former patients and made adjustments for local context and language usage. Finally the reliability of the questionnaire was verified using the test re-test methodology on 36 consenting participants. The questionnaire was repeated and answers compared within 2–4 weeks following the baseline visit. In each case the interview was conducted by the same interviewer who administered the first questionnaire. Scores from the first interview were compared to those from the second interview using Cohen’s kappa coefficient (k). The coefficient ranged from 0.7030 to 1.000 indicating substantial to perfect agreement except for one question with a k of 0.5567 which indicates moderate agreement. (The questionnaire is provided as supplementary information, S1 Text). The survey was completed via one-on-one, face-to-face interviews between participants and a trained, multi-lingual study interviewer in a private room. Interviews were conducted in English and in the mother tongue of participants as required. Patients were grouped by stage at diagnosis as early (0-II) or advanced (III-IV). We compared the patient groups with respect to demographic, socioeconomic and clinical characteristics and responses to the Barriers to Care questionnaire. From the ‘barrier to care’ questionnaire data, we categorized women by time between awareness of a breast symptom and first visit to a health care facility and by the types of health care facilities they visited prior to CHBAH: self-referral directly to CHBAH or after a primary care clinic visit or referral from a primary care clinic to a secondary care hospital before CHBAH. We also grouped patients by the number of visits to each type of facility.

### Statistical analysis

We used the Pearson chi squared and Fisher’s exact tests to examine differences in the proportion of early/advanced stage between categorical variables. We computed means and medians for continuous variables and used Student’s t-test and the Wilcoxon rank-sum test to determine differences between groups in means and medians, respectively. To examine associations with advanced stage, we then developed multivariable logistic regression models of factors. We included in the multivariate models variables for which p-values were <0.1 in bivariate analysis. ORs were examined in 4 models by adding in a stepwise fashion, factors of influence (for socio-demographic, clinical and lifestyle, knowledge and awareness, and health system) to examine the association between these factors and advanced presentation of breast cancer. Analysis was performed using Stata version 14 (StataCorp Ltd, Texas, USA).

## Results

Of the 499 enrolled women, 243 (48.7%) were diagnosed with early-stage disease (0-II), including 11 (4.5%) with in-situ cancers and 256 (51.3%) with advanced-stage disease (III-IV). Characteristics of the cohort are summarized in Table 1.

Although age overall was not associated with advanced stage at diagnosis, women <40 years had a higher risk of diagnosis at an advanced stage than women >40 years (p = 0.013). Other factors that initially appeared to be associated with advanced-stage disease were no high school education, lower household socio-economic status, higher parity, lower score on self-reported knowledge and awareness of breast cancer, no hypertension, and HIV co-infection.

Table 2 describes the association of personal delays and health system factors with advanced-stage diagnosis.

**Table 2:** Descriptive statistics for patients presenting with early compared with late stage breast cancer at CHBAH breast clinic that experienced personal and/or health system delays.

	Total	Early stage	Late stage	ρ-	Chi-square
		(0–11)	(III & IV)	value	Statistic/DF
	N = 499 (%)	N = 243 (%)	N = 256 (%)		
**Time to first presentation to HS**					
<1 month	307 (61.5)	173 (71.2)	134 (52.3)	**<0.001**	22.881
1–3 months	64 (12.8)	30 (12.3)	34 (13.3)		DF = 2
>3 months	128 (25.7)	40 (16.5)	88 (34.4)		
**Residential distance from CHBAH**					
< 10km	206 (41.3)	105 (43.2)	101 (39.5)	0.328	2.2277
10- 20km	104 (20.8)	54 (22.2)	50 (19.5)		DF = 2
>20km	189 (37.9)	84 (34.6)	105 (41.0)		
**Number of HS visits before reaching CHBAH**					
0–1	257 (51.5)	145 (59.7)	112 (43.8)	**0.002**	12.661
2	73 (14.6)	30 (12.3)	43 (16.8)		DF = 2
≥3	169 (33.9)	68 (28.0)	101 (39.4)		
**Referral mode to CHBAH**					
Self + primary–direct	323 (64.7)	166 (68.3)	157 (61.3)	0.103	2.639
Primary/secondary–indirect	176 (35.3)	77 (31.7)	99 (38.7)		DF = 1
**Self/primary (direct)**	**323 (64.7)**	**166 (68.3)**	**157 (61.3)**	**<0.001**	15.439
0–1 visits–self-primary	251 (77.7)	143(86.1)	108 (68.8)		DF = 2
2 visits self/primary	49 (15.2)	18 (10.9)	31 (19.7)		
≥3 visits self/primary	23 (7.1)	5 (3.0)	18 (11.5)		
**Primary/secondary (indirect)**	**176 (35.3)**	**77 (31.7)**	**99 (38.7)**	0.716	0.6618
0-1visits primary/secondary	6 (3.4)	2 (2.6)	4 (4.1)		DF = 2
2 visitsprimary/secondary	24 (13.6)	12 (15.6)	12 (12.1)		
≥3 visitsprimary/secondary	146 (83.0)	63 (81.8)	83 (83.8)		

Women who reported a delay of >1 month between identifying breast symptoms and their first visit to the health system were more likely than those who made the first visit sooner to be diagnosed at a late stage (p< 0.001). As shown in Table 3, the two most commonly stated reasons for delays were fear of diagnosis or treatment and failure to recognize that breast symptoms were serious. Other reasons were conflicting commitments, such as caring for sick family members and transport problems.

**Table 3:** Reasons for personal delays into the health system.

	Staging at diagnosis					
Reasons for delay	Early stage		Late stage		Total	
	N = 243	%	N = 256	%	N = 499	%
No delay	173	71.19	136	53.13	309	61.92
Fear of diagnosis	13	5.35	20	7.81	33	6.61
Thought it was minor ailment	56	23.05	96	37.50	152	30.46
No one to look after the children	0	0.00	3	1.17	3	0.60
Worried no money for treatment	1	0.41	1	0.39	2	0.40
**Total**	**243**	**100.00**	**256**	**100.00**	**499**	**100.00**

Most participants (64.7%) referred themselves to CHBAH or were referred directly by a primary care clinic or a private general practitioner, bypassing the secondary hospitals. Among those patients, more than half had only one visit prior to diagnosis, and those with more visits before reaching CHBAH were more likely to be diagnosed at a late stage (ρ <0.001). Among patients seen at secondary hospitals, more than 80% had at least 3 visits prior to diagnosis and the actual number of visits had no further impact on stage at presentation. Repeat visits within the referral system appear to have been caused by failure to diagnose and delays with appointments and test results. The details are provided as supporting information S2 Table.

In the bivariate analysis Table 4, those who completed high school/ any tertiary education were at lower risk for advanced -stage presentation (OR  =  0.60, 95%CI: 0.40–0.88) than those who had informal or only primary school education. For every unit increase in parity there was a borderline higher risk of advanced stage presentation, (OR 1.10, 95%CI: 0.99–1.21). Those treated for hypertension were found to be at lower risk for advanced stage at diagnosis (OR = 0.59, 95%CI: 0.41–0.85) as were those with greater knowledge and awareness of breast cancer (OR = 0.82, 95%CI: 0.74–0.91). Patients aged <40 years (OR = 1.93, 95%CI: 1.05–3.58) and those with luminal B and triple negative breast cancer subtypes (OR = 1.86, 95%CI: 1.10–3.14 and OR = 2.61, 95%CI: 1.69–5.30 respectively) were at higher risk for advanced stage at diagnosis than those with a luminal A subtype. Those who reported having taken >3 months after noting a breast symptom to visit a healthcare facility (OR  =  2.84, 95%CI: 1.84–4.39) were also at higher risk for advanced-stage disease at diagnosis than patients who had taken less than one month. Those who had more than one visit prior to reaching CHBAH also were more likely to be diagnosed at an advanced stage than patients who had no more than one visit (mainly those who had self and direct clinic referral to CHBAH).

**Table 4:** Bivariate analysis of variables associated with late stage presentation amongst patients diagnosed with breast cancer at the CHBAH Breast Clinic from 2014–2016.

Variables	Bivariate analysis	
	OR (95% CI)	P-value*
**SOCIO-DEMOGRAPHIC CHARACTERISTICS**		
**Age**		
< 40	1.93 (1.05–3.58)	**0.0120**
40–49	1.10 (0.67–1.82)	
50–59	Reference	
60–69	0.62 (0.37–1.07)	
70 and above	1.01 (0.58–1.77)	
**Marital status**		
Single	1.10 (0.70–1.72)	0.7358
Married/co-habiting	Reference	
Divorced/widowed	0.91 (0.61–1.37)	
**Level of education**		
Completion of Informal / Primary	Reference	**0.0096**
Completion of High school/ any tertiary education	0.60 (0.40–0.88)	
**Household socioeconomic status**	0.90 (0.82–0.98)	**0.0214**
**Employment status**		
Unemployed	Reference	0.2433
Employed	1.43 (0.93–2.19)	
Retired	1.06 (0.69–1.63)	
**Parity**	1.10 (0.99–1.21)	**0.0624**
**CLINICAL AND LIFESTYLE FACTORS**		
**BMI**	0.99 (0.97–1.01)	0.3353
**Smoking**		
No	Reference	
Yes	0.61 (0.30–1.23)	0.164
**Alcohol consumption**		
No	Reference	
Yes	0.94 (0.60–1.45)	0.7707
**Hypertension**		
No	Reference	
Yes	0.59 (0.41–0.85)	**0.0047**
**HIV Status**		
Negative	Reference	
Positive	1.72 (1.12–2.65)	**0.1125**
**Intrinsic receptor subtype–IHC**		
Luminal A	Reference	
Luminal B	1.86 (1.10–3.14)	**0.0450**
HER2 enriched	1.77 (0.77–4.07)	
Triple negative	2.61 (1.69–5.30)	
**KNOWLEDE/AWARENESS SCORE (0–9)**	0.82 (0.74–0.91)	**0.0001**
**HEALTH SYSTEM FACTORS**		
**Prior clinical breast examination**		
Clinician	Reference	
None/self	1.33 (0.52–3.43)	0.5531
**Clinic waiting times barrier to attendance**		
No	Reference	
Yes	0.77 (0.41–1.45)	0.411
**Time to first presentation to health system**		
<1 month	Reference	**<0.001**
1–3 months	1.46 (0.85–2.51)	
>3 months	2.84 (1.84–4.39)	
**Residential distance from CHBAH**		
< 10km	Reference	0.3278
10- 20km	0.96 (0.6–1.5)	
>20km	1.3 (0.9–1.9)	
**Referral HS visits prior to CHBAH**		
0–1	Reference	**0.0017**
2	1.86 (1.10–3.14)	
≥3	1.92 (1.30–2.85)	
**Referral mode to CHBAH**		
Self + primary–direct route	Reference	0.1023
Primary/secondary–indirect route	1.36 (0.94–1.97)	

*Variables at p<0.1 on bivariate analysis are presented in boldface and were considered in the multiple logistic regression model analysis.

IHC: Immunohistochemistry.

Associations significant at the bivariate level were further explored in Table 5 in individual and combined multiple logistic regression models 1–4 and 5–7 respectively.

**Table 5:** Multiple logistic regression models of factors influencing late stage disease presentation among patients diagnosed with breast cancer at CHBAH Breast Clinic 2014–2016.

Multiple logistic regression models								
Variables	Bivariate analysis OR (95% CL)	Model 1 Sociodemo-graphic (SD) OR (95% CL)	Model 2 Clinical & Lifestyle (CL) OR (95% CI)	Model 3 Knowledge & Awareness (K) OR (95% CI)	Model 4 Health System (H) OR (95% CI)	Model 5 SD +CL OR (95% CI)	Model 6 SD + K OR (95% CI)	Model 7 SD+CL+HS OR (95% CI)
**SOCIO-DEMOGRAPHIC**								
**Age**								
< 40	**1.93 (1.05–3.58)**	**2.16 (1.09–4.27)**				1.77 (0.84–3.70)	1.81 (0.86–3.83)	1.74 (0.80–3.80)
40–49	1.10 (0.67–1.82)	1.30 (0.76–2.23)				1.08 (0.60–1.94)	1.10 (0.61–2.00)	1.02 (0.55–1.89)
50–59	Referent	Referent				Referent	Referent	Referent
60–69	0.62 (0.37–1.07)	0.57 (0.32–0.99)				0.66 (0.35–1.24)	0.65 (0.34–1.24)	0.79 (0.40–1.54)
70 and above	1.01 (0.58–1.77)	0.78 (0.42–1.44)				0.93 (0.47–1.86)	0.90 (0.45–1.81)	1.03 (0.50–2.16)
**Education level**								
Informal/primary	Referent	Referent				Referent	Referent	Referent
≥ High school	**0.60 (0.40–0.88)**	**0.52 (0.33–0.82)**				**0.52 (0.32–0.85)**	**0.57 (0.34–0.95)**	**0.59 (0.35–1.00)**
**Household socioeconomic**	**0.90 (0.82–0.98)**	0.94 (0.85–1.04)				0.96 (0.84–1.09)	0.99 (0.87–1.13)	0.99 (0.87–1.14)
**Parity**	1.10 (0.99–1.21)	1.12 (1.00–1.25)				1.11 (0.98–1.26)	1.11 (0.97–1.26)	1.13 (0.98–1.29)
**CLINICAL & LIFESTLE**								
**HIV status**								
Negative	Referent		Referent			Referent	Referent	Referent
Positive	**1.72 (1.12–2.65)**		1.48 (0.92–2.36)			1.49 (0.88–2.51)	1.47 (0.87–2.48)	1.53 (0.89–2.65)
**Treated hypertension**								
No	Referent		Referent			Referent	Referent	Referent
Yes	**0.59 (0.41–0.85)**		0.68 (0.46–1.02)			0.69 (0.43–1.10)	0.66 (0.41–1.06)	0.63 (0.38–1.04)
**BMI** (continuous)	0.99 (0.97–1.01)		1.00 (0.97–1.02)			1.00 (0.97–1.02)	0.99 (0.97–1.02)	1.00 (0.97–1.03)
**Intrinsic receptor subtype**								
Luminal A	Referent		Referent			Referent	Referent	Referent
Luminal B	**1.86 (1.10–3.14)**		**1.93 (1.10–3.39)**			1.75 (0.96–3.17)	1.80 (0.99–3.29)	**2.25 (1.18–4.29)**
HER2-enriched	1.77 (0.77–4.07)		1.48 (0.92–2.36)			1.30 (0.51–3.33)	1.34 (0.52–3.45)	1.34 (0.50–3.59)
Triple negative	**2.61 (1.69–5.30)**		**3.17 (1.48–6.80)**			**2.67 (1.20–5.95)**	**2.73 (1.21–6.15)**	**3.17 (1.36–7.43)**
**KNOWLEDGE & AWARENESS**								
**Score (0–9)**	**0.82 (0.74–0.91)**			**0.82 (0.74–0.91)**			**0.84 (0.75–0.94)**	**0.86 (0.76–0.97)**
**HEALTH SYSTEM FACTORS**								
**Time to 1**^**st**^ **visit to health system**								
< 1 month	Referent				Referent			Referent
1–3 months	1.46 (0.85–2.51)				1.58 (0.91–2.74)			1.63 (0.87–3.06)
> 3 months	**2.84 (1.84–4.39)**				**3.14 (2.00–4.92)**			**2.79 (1.66–4.70)**
**Number of HS visits prior to CHBAH**								
0–1	Referent				Referent			Referent
2	**1.86 (1.10–3.14)**				**2.53 (1.41–4.53)**			**3.19 (1.64–6.23)**
≥ 3	**1.92 (1.30–2.85)**				**3.52 (1.73–7.16)**			**2.73 (1.22–6.12)**
**Referral pathway to CHBAH**								
Self & primary	Referent				Referent			Referent
Primary + secondary	1.36 (0.94–1.97)				0.53 (0.27–1.03)			0.63 (0.30–1.33)
**Pseudo R**^**2**^		**0.0426**	**0.0332**	**0.0234**	**0.0608**	**0.0646**	**0.0803**	**0.1279**

Socio-demographic factors (model 1) independently explained 4% of the variance and were shown to influence the likelihood of advanced stage at diagnosis in part by younger age and decreased level of education. Clinical and lifestyle factors (model 2) explained 3% of the variance, with Luminal B and Triple Negative having greater odds of advanced-stage presentation than Luminal A subtype. Greater knowledge and awareness of breast cancer had a lower odds for advanced-presentation (OR 0.82, 95%CI 0.74–0.91) and explained just over 2% of the variance (model 3). Health system factors (model 4) explained 6% of the variance with patient delays >3 months having more than 3 times greater odds for advanced stage–presentation. In addition patients experiencing >2 referral health system visits had greater than twice the odds for advanced-stage presentation compared with those having 0 visits (self-referrals) and 1 visit (within the primary health system). In the final model with all factors included (model 7), 13% of the variance was explained. Notably, the associations of advanced stage with lower education, tumour subtype, and health system factors were not attenuated upon adjustment; the association with a lower knowledge score was slightly attenuated, whereas associations with household socioeconomic status, parity, HIV, and treated hypertension were no longer significant in mutually adjusted models. Thus, although HIV+ women tended to present at more advanced stages, because they were younger there was some attenuation of this association upon adjustment for either of these factors.

A cluster analysis was undertaken to distinguish group associations of sociodemographic variables with time to breast cancer disease presentation. The analysis was performed on continuous variables, omitting correlating variables, namely age, self-reported parity, self-reported household SES score (0–6) (based on home ownership, car ownership, hot and cold running water in home, flush toilet in house, owning a microwave, owning a washing machine) and the self-reported knowledge score (0–9). Three clusters were generated using the K mean and an ANOVA was performed to identify any statistical differences in the means between the 3 clusters and stage at diagnosis. The results are tabulated in the supporting information S3 Table and reveal no statistical differences between the three groups.

## Discussion

In our cohort of predominantly black women from socioeconomically disadvantaged urban communities around Johannesburg, patients with less than a high school education, those with little awareness or knowledge of breast cancer, those with luminal B and triple negative breast cancer subtypes, those who took more than three months from first recognition of their breast symptoms to visit a health care facility, and those who experienced more than 2 visits within the primary and secondary health system referral network were more likely to have been diagnosed at an advanced stage. We attribute the delay in making the first visit mainly to the two extremes of either patient lack of awareness of symptoms or fear of diagnosis. However, the multiple visits due to healthcare system failure to diagnose or inefficiency in scheduling appointments and retrieving laboratory results were major contributors to diagnostic delays and markedly impacted patient outcomes, especially those with aggressive receptor subtypes. More than 90% of the cohort reported receiving no routine clinical breast examinations during routine visits to women’s health and other clinics prior to experiencing their breast symptoms. Thus clinical breast examination services provided by trained doctors and nurses, though affordable, are not a standard feature of the primary health care system in SA.

Additionally, we found that HIV+ women tended to have more advanced stage. This was in part due to their younger age, but HIV+ women can be reached through their existing contacts with the health system. Education on breast awareness could be included when raising awareness of women’s cancer issues in HIV+ women. Although breast cancer is not a HIV-associated malignancy, as it is a common cancer in women in general, it remains a relevant issue in HIV-affected women too.

According to other studies, lack of knowledge and awareness of breast cancer and symptoms and fear of diagnosis and treatments are widespread and associated with advanced-stage diagnosis in other LMICs. Geographical isolation, inadequate financial resources, a preference for consulting traditional healers linked with cultural and religious beliefs, and a shortage of adequately trained healthcare professionals and facilities to diagnose and treat cancer are also widespread. Most LMICs also have limited cancer prevention and control policies and cancer registries able to monitor incidence and mortality [4, 12, 15, 23, 27, 28, 30, 31, 61].

In HICs, routine cancer screening is available, and healthcare services are well resourced. Yet in the USA, the breast cancer mortality rate is disproportionately high among blacks due to more advanced stage at diagnosis (after controlling for the higher proportion of triple negative subtype). Lack of knowledge of symptoms and risk factors, fear of treatments, higher financial burden of treatments, more lifestyle-associated risk factors and comorbidities, cultural issues, lack of partner support, stigma and taboo, and inferior health care services may all be associated with delays in accessing treatment[2, 24, 30, 32]

Our findings are based on a sample of patients from an urban black community in and around Johannesburg and may therefore have limited generalizability. Our information about delays in care seeking and numbers and types of healthcare visits is based on patient survey responses, rather than administrative data, which may be biased or less accurate. With a near 50% late stage disease in a sample size of 500 women, we had 80% power to detect ORs of 1.66 or larger for exposure prevalence of 50% and an OR of 2.08 or larger for rarer exposure prevalence of 10%. Larger studies will be required to detect smaller ORs. Findings were consistent with other data supporting associations with stage at diagnosis.

Fortunately, many of these factors are modifiable. Partnering with Cancer NGOs in SA, coordinating outreach programs at community and clinic levels to increase knowledge and awareness of breast cancer symptoms and risk factors, allaying fears of diagnosis and treatments, and active navigation of patients through the referral networks would undoubtedly enable more patients to be diagnosed and treated at an early stage. In addition, ongoing education and advisory support for primary and secondary tier doctors and nurses supported by the implementation of efficient referral algorithms would improve breast cancer detection and prevent unnecessary delays within the referral network.

## Conclusion

Advanced-stage diagnosis of breast cancer among urban SA communities relying on public healthcare services is associated with patient- and health system-related barriers to care. Community and healthcare worker awareness and education programs, swift diagnostic workups and expedited referral processes may overcome these barriers and reduce breast cancer morbidity and mortality.

## Supporting information

S1 Table (XLS)

S2 Table (XLSX)

S3 Table (DOCX)

S1 Text (DOCX)
