# Supplementary material for: Barriers to early presentation of breast cancer among women in Soweto, South Africa
Source: PLoS One. 2018 Feb 2;13(2):e0192071. doi: 10.1371/journal.pone.0192071 (PMC5796726; doi:10.1371/journal.pone.0192071)
Supplement: S3 Table — (DOCX) [file pone.0192071.s003.docx]

**S3 Table**

**Cluster analysis to identify group associations of socio-demographic variables with time to breast cancer disease presentation**

| \|  \|  \| **Clusters** \| \| \| \| \| --- \| --- \| --- \| --- \| --- \| --- \| \| **^a^Sociodemographic**  **Parameters selected** \|  \| ^b^1 (n=103) \| ^c^2 (n=176) \| ^d^3 (n=192) \| Total (n=471) \| \| **Age on presentation (years)** \| Minimum  Mean  Maximum \| 67  76.1  96 \| 26  41.3  50 \| 50  58.2  68 \| 26  55.8  96 \| \| **Parity** \| Minimum  Mean  Maximum \| 0  4.4  10 \| 1  2.4  8 \| 0  2.8  6 \| 0  3.0  10 \| \| **Household SES (Total=6)** \| Minimum  Mean  Maximum \| 0  3.9  6 \| 0  3.6  6 \| 0  3.8  6 \| 0  3.7  6 \| \| **Knowledge score (Total=9)** \| Minimum  Mean  Maximum \| 0  5.3  9 \| 1  6.2  9 \| 1  5.9  9 \| 0  5.9  9 \| |  |  |  |  |  |
| --- | --- | --- | --- | --- | --- | --- | --- | --- | --- | --- | --- | --- | --- | --- | --- | --- | --- | --- | --- | --- | --- | --- | --- | --- | --- | --- | --- | --- | --- | --- | --- | --- | --- | --- | --- | --- | --- | --- | --- | --- | --- |

**ANOVA performed on cluster means produced an F-statistic = 1.43 and ρ = 0.2410**

^a^ Correlating variables were omitted and continuous variables were selected for the clustering analysis

^b^ Cluster 1 characteristics: old women with high parity, moderate household possession score (Household SES) and low to moderate knowledge and awareness of breast cancer

^c^ Cluster 2 characteristics: young women with low parity, low household SES scores and moderate knowledge and awareness of breast cancer

^d^ Cluster 3 characteristics: middle age women with moderate parity, moderate household SES scores and moderate knowledge and awareness of breast cancer
