# Supplementary material for: Barriers to early presentation of breast cancer among women in Soweto, South Africa
Source: PLoS One. 2018 Feb 2;13(2):e0192071. doi: 10.1371/journal.pone.0192071 (PMC5796726; doi:10.1371/journal.pone.0192071)
Supplement: S1 Text — (DOCX) [file pone.0192071.s004.docx]

**S1 Text**

**Barriers to ACCESS AND BREAST HEALTH Care questionnaire**

**Awareness and knowledge of breast cancer**

1. Do you personally know somebody with breast cancer?

🞊 Yes 🞊 No 🞊Not sure/don’t know

1. Have you heard anything about breast cancer before?

🞊 Yes 🞊 No 🞊Not sure/don’t know

*To the following statements please select the option which most closely applies to you.*

1. A person can catch breast cancer from someone else.

🞊Strongly disagree 🞊Disagree 🞊Unsure/neutral 🞊Agree 🞊Strongly agree

1. Cancer can run in families and be inherited

🞊Strongly disagree 🞊Disagree 🞊Unsure/neutral 🞊Agree 🞊Strongly agree

1. Breast cancer can be caused by an injury to the breast

🞊Strongly disagree 🞊Disagree 🞊Unsure/neutral 🞊Agree 🞊Strongly agree

1. Breast cancer can be caused by a curse

🞊Strongly disagree 🞊Disagree 🞊Unsure/neutral 🞊Agree 🞊Strongly agree

1. A painless lump can be a sign of breast cancer

🞊Strongly disagree 🞊Disagree 🞊Unsure/neutral 🞊Agree 🞊Strongly agree

1. A painful breast can be a sign of breast cancer

🞊Strongly disagree 🞊Disagree 🞊Unsure/neutral 🞊Agree 🞊Strongly agree

1. Fluid coming from the nipple can be a sign of breast cancer

🞊Strongly disagree 🞊Disagree 🞊Unsure/neutral 🞊Agree 🞊Strongly agree

**Personal hurdles to seeking help**

1. You thought it was a minor ailment (nothing serious)

🞊Strongly disagree 🞊Disagree 🞊Unsure/neutral 🞊Agree 🞊Strongly agree

1. You were afraid you would be diagnosed with a serious problem

🞊Strongly disagree 🞊Disagree 🞊Unsure/neutral 🞊Agree 🞊Strongly agree

**Family, community and cultural hurdles to seeking help**

1. You had to get permission from your partner/husband to go to the clinic/hospital

🞊Strongly disagree 🞊Disagree 🞊Unsure/neutral 🞊Agree 🞊Strongly agree

1. There was no one at home to look after the children/grandchildren

🞊Strongly disagree 🞊Disagree 🞊Unsure/neutral 🞊Agree 🞊Strongly agree

1. You were scared of rejection by your family

🞊Strongly disagree 🞊Disagree 🞊Unsure/neutral 🞊Agree 🞊Strongly agree

1. When you don’t feel well your friends and community are supportive

🞊Strongly disagree 🞊Disagree 🞊Unsure/neutral 🞊Agree 🞊Strongly agree

1. A traditional healer e.g. Sangoma may be of help with breast problems

🞊Strongly disagree 🞊Disagree 🞊Unsure/neutral 🞊Agree 🞊Strongly agree

1. Faith healing may be of help with breast problems

🞊Strongly disagree 🞊Disagree 🞊Unsure/neutral 🞊Agree 🞊Strongly agree

**Economic and geographic barriers to seeking help**

1. Are you employed?

🞊Yes 🞊No

1. You are worried you may not have enough money for treatment

🞊Strongly disagree 🞊Disagree 🞊Unsure/neutral 🞊Agree 🞊Strongly agree

1. You are worried you may lose income whilst being treated

🞊Strongly disagree 🞊Disagree 🞊Unsure/neutral 🞊Agree 🞊Strongly agree

1. You live far away from the hospital or clinic and so you are delayed

🞊Strongly disagree 🞊Disagree 🞊Unsure/neutral 🞊Agree 🞊Strongly agree

**Health system barriers to early stage presentation of disease**

1. In the past before you had a breast problem, did anyone ever examine your breasts?

🞊Yes 🞊No

If yes, which provider examined your breasts?

🞊 Doctor 🞊 Nurse 🞊Self examined 🞊Other _________________________________

1. Have you ever had a breast ultrasound/mammogram/x-ray before you were diagnosed?

🞊Yes 🞊No 🞊Not sure

*To the following statements please select the option which most closely applies to you.*

1. The waiting time in the clinics is a reason not to go there

🞊Strongly disagree 🞊Disagree 🞊Unsure/neutral 🞊Agree 🞊Strongly agree

1. Hospitals are scary to me and I struggle to find my way around

🞊Strongly disagree 🞊Disagree 🞊Unsure/neutral 🞊Agree 🞊Strongly agree

1. The clinics I have attended have provided me with satisfactory services for my breast problem

🞊Strongly disagree 🞊Disagree 🞊Unsure/neutral 🞊Agree 🞊Strongly agree

**Navigation through the referral health system**

1. Who did you first tell after you found/noticed a change in your breast?
2. Family member/member of my household
3. Chemist/pharmacist
4. Neighbour or friend
5. Home based community worker
6. Private doctor (GP)
7. Sangoma/ traditional healer/inyanga
8. Community clinic
9. Homeopath or herbalist
10. Hospital nursing sister/doctor
11. Faith healer
12. Church pastor/elder
13. Social worker/counselor

*Interviewer please probe delay period from time first noticed breast problem to first contact with the health system. For delays >1 month, if the patient delayed “I delayed” must be clicked. If the health system delayed >1 month ”Health System delayed”must be clicked.*

Visit 1

Who did you first see

🞊Private doctor 🞊Clinic/primary health centre 🞊Referral hospital 🞊Tertiarty breast clinic 🞊 Sangoma/traditional healer/inyanga 🞊 Faith healer 🞊Homeopath/herbalist 🞊 Church pastor/elder 🞊Chemist/pharmacist 🞊 Home community care worker 🞊 Social worker/counselor 🞊Other

Time to delay 🞊weeks 🞊months 🞊 years

Visit details

If delay greater than 1 month, if patient delayed: I delayed because of

Fear of diagnosisFear of treatmentNeighbour / friendNo transport money

Partner forbade meFear of rejection by familyNo one to care for the children

Didn’t think it was seriousNot applicableOther

If delay greater than 1 month, if Health System delayed: Health System delayed because of

They gave me pills and told me to come backThere was a delay for test results

There was a waiting list to get an appointmentThe sister / doctor turned me away

The doctor / clinic did not think it was seriousNot applicableOther

**Repeated for each subsequent visit completed**

## 
